# Diagnostic value of multiple projection angle X-ray and CT 3D reconstruction for long-term unreduced posterior hip dislocation

**DOI:** 10.3389/fsurg.2025.1512955

**Published:** 2025-05-08

**Authors:** Yansong Liu, Yongbo Ma, Zeming Liu, Xuzhuang Ding, Xiaowei Yao, Jiangqi Chang, Hao Li, Tao Wu

**Affiliations:** ^1^ Department of Orthopaedic Surgery, The Third Hospital of Hebei Medical University, Shijiazhuang, Hebei, China; ^2^ Department of Orthopaedic Surgery, Hebei Provincial Chest Hospital, Shijiazhuang, Hebei, China

**Keywords:** aureole sign, diagnostic value, hip joint, radiography, rhombus sign

## Abstract

**Background:**

Long-term unreduced posterior hip dislocation is a rare and diagnostically challenging condition, with imaging findings often indistinguishable from those of other end-stage hip diseases. It remains a great challenge to determine whether certain imaging characteristics can improve the clinical diagnosis rate of long-term unreduced posterior hip dislocation.

**Methods:**

We retrospectively reviewed 24 patients from 2010 to 2022. The diagnostic values of multiple projection angle X-ray and CT 3D reconstruction for long-term unreduced posterior hip dislocation were evaluated.

**Results:**

For aureole sign, 45.83% of patients (sensitivity = 45.83%, specificity = 81.52%, accuracy = 78.67%, Youden's index = 0.274, positive predictive value (PPV) = 17.74%, negative predictive value (NPV) = 94.54%, intraobserver consistency = 0.930, and interobserver consistency = 0.903) were diagnosed correctly. For obturator oblique radiograph of the pelvis, 58.33% of patients (sensitivity = 58.33%, specificity = 82.25%, accuracy = 80.33%, Youden's index = 0.406, PPV = 22.22%, NPV = 95.78%, intraobserver consistency = 0.923, and interobserver consistency = 0.900) were diagnosed correctly. For rhombus sign, 70.83% of patients (sensitivity = 70.83%, specificity = 90.94%, accuracy = 89.33%, Youden's index = 0.618, PPV = 40.48%, NPV = 97.29%, intraobserver consistency = 0.943, and interobserver consistency = 0.900) were diagnosed correctly. For CT 3D reconstruction, axial CT (sensitivity = 70.83%), coronal multiplanar reconstruction (sensitivity = 58.33%), and sagittal multiplanar reconstruction (sensitivity = 54.17%), all had high diagnostic values.

**Conclusions:**

The signs, projection angle X-ray, and CT 3D reconstruction identified in this study are valuable in improving the diagnosis for long-term unreduced posterior hip dislocation.

## Introduction

Long-term unreduced posterior hip dislocation is an uncommon type of dislocation, which, to our best knowledge, is reported in developing countries with only a few cases documented (1–3). The pathogenesis of posterior hip dislocation principally stems from two major factors: traumatic events, typically resulting from traffic accidents, and underlying conditions, particularly developmental dysplasia of the hip (DDH) (2, 4). Patients often present with hip pain and limited movement (5), which are consistent with the presentation of end-stage hip disease. Because this disease is difficult to distinguish on radiography, orthopedists often ignore this disease (6) or misdiagnose patients with long-term unreduced posterior hip dislocation with ARCO stage IV aseptic necrosis of the femoral head or Crowe stage IV DDH and perform conventional total hip arthroplasty (THA) surgical treatment. Patients with traumatic hip dislocations who undergo timely intervention, such as effective closed reduction within 3 weeks, generally achieve favorable outcomes. Consequently, the progression to long-term unreduced posterior hip dislocation is exceptionally rare. Long-term unreduced traumatic dislocation often affects subsequent hip function and leads to gait abnormalities, such as a limping gait. However, due to neglect of the condition and poor medical care, it is difficult for a considerable number of patients to receive timely and appropriate treatment, which leads to long-term dislocation, especially posterior dislocation of the hip.

Research on long-term unreduced posterior hip dislocation remains limited. Liu et al. (7) reported that the two specialty signs—the aureole sign on X-ray and the rhombus or “I” sign on CT—are crucial for diagnosing this condition. Patients typically undergo an anteroposterior X-ray of the pelvis, with CT imaging used as a supplementary tool when needed. However, relying solely on X-ray and CT scans can result in misdiagnosis in approximately 50% of cases (7).

This highlights a critical gap in the accurate preoperative diagnosis of this condition. Therefore, this study aims to identify additional imaging signs or optimal projection angles on X-ray and CT that could enhance diagnostic accuracy. Specifically, we designed a four-part experiment to compare the diagnostic value of various X-ray signs, anteroposterior radiographs, different X-ray projection postures, axial CT signs, and different CT reformation techniques in diagnosing long-term unreduced posterior hip dislocation. The primary research question of this study is to determine which imaging methods provide the most accurate preoperative diagnosis of this rare and challenging condition.

## Methods

### Study population

A retrospective review was conducted on 24 patients diagnosed with long-term unreduced posterior hip dislocation (dislocation group) and 276 patients diagnosed with severe hip osteoarthritis (OA) (control group) between 2010 and 2022. A total of 300 patients were enrolled in the study. The inclusion criteria for the dislocation group included patients with an interval of >1 year between the injury and the initial visit, with one side injured and the other side normal (8, 9). Patients with a diagnosis of posterior dislocation of the hip joint of <1 year (8, 9) or had long-term unreduced posterior hip dislocations bilaterally; those with developmental dysplasia of the hip (DDH), identified based on femoral morphology and other distinguishing characteristics; and those with a history of hip dislocation during childhood were excluded from the study. Initial sample size calculation: The number of patients required for an overall incidence (*π*) of 0.01 and a test efficacy (power) of 0.9 was 230. For ease of calculation, a total of 276 patients were included as controls in this study.

It is important to note that the “normal” diagnostic test requires a distinction between “patients” and “healthy people” in particular. However, the disease has obvious signs and symptoms, and significant pathological changes can be observed on imaging, so the clinical omission is not due to confusing patients with the healthy population but rather to failure to distinguish long-term unreduced posterior hip dislocation from other common causes of severe end-stage hip disease (e.g., severe hip osteoarthritis). Therefore, the control group selected for this trial was not a healthy population but a population with other severe end-stage hip diseases. Therefore, all the data (sensitivity, specificity, accuracy, etc.) obtained from the experiment represent the ability of the imaging signs and diagnostic methods to differentiate between the “dislocation group (long-term unreduced posterior hip dislocation)” and the “control group,” rather than the ability to differentiate from the healthy population.

The study was approved by the Institutional Review Board of the Third Hospital of Hebei Medical University and was conducted in accordance with the Declaration of Helsinki and the Health Insurance Portability and Accountability Act (HIPAA). This was a retrospective study, and data on basic patient information, medical history, history of injury, and information on concomitant injuries were obtained from the admission records. Previous visits were recorded when each patient was hospitalized at our hospital, and there was no need to contact patients repeatedly. Although a retrospective study design was used, potential bias, such as selection bias or recall bias, may have been introduced. All patient information was confirmed prior to data analysis, and written consent was obtained only for those patients whose images were released. Patient data were anonymized to protect confidentiality and ensure compliance with ethical guidelines.

### Evaluation of diagnostic methods

The purpose of our study is to determine some diagnostic signs and imaging angles that can improve the accuracy of the initial diagnosis of long-term unreduced posterior hip dislocation. In terms of X-ray examination, we compared the diagnostic values of different X-ray signs on anteroposterior pelvic radiographs and different X-ray projection postures in diagnosing long-term unreduced posterior hip dislocation. In terms of CT examination, we compared the diagnostic values of axial CT signs and different types of CT reformation techniques in diagnosing long-term unreduced posterior hip dislocation. The diagnosis was made by observing all the pelvic anteroposterior X-ray and axial CT in the experiment, respectively, by two observers without any information. Their observations were recorded, and the experimental results were named blank group (X-ray) and blank group (CT).

**Figure 1:**
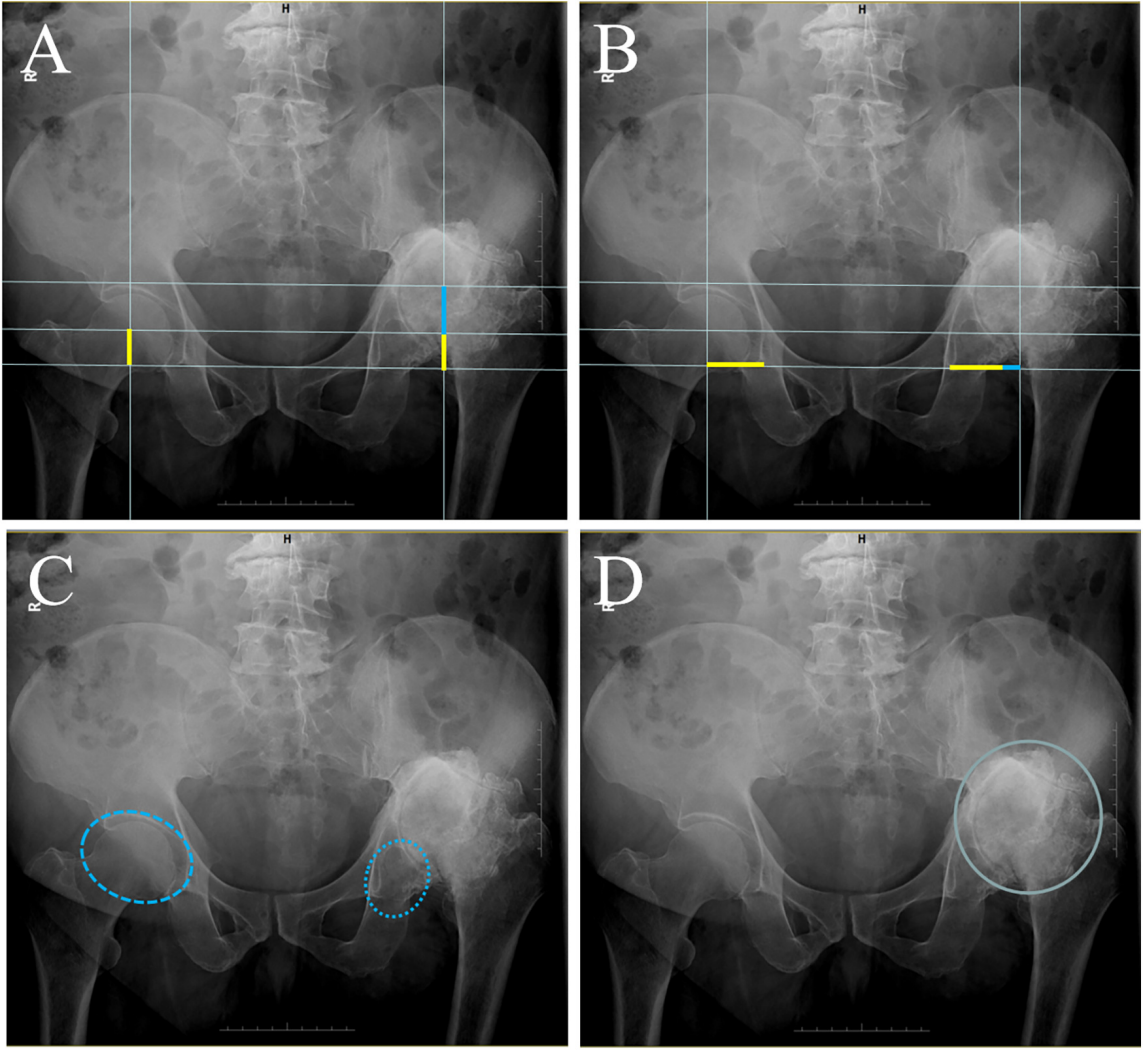
Anterior posterior view of X-ray radiological characteristics in patients with long-term unreduced hip joint dislocation. **(A)** The anterior posterior pelvic radiograph of the patient was placed on a standard grid line, and the vertical distance of the femoral head rotational center to the ipsilateral teardrop was measured bilaterally. The yellow lines indicate the distance of the femoral head rotational center on the healthy side to the ipsilateral tear drop in the vertical direction, and the blue lines indicate the portion of the femoral head rotational center on the affected side to the vertical distance to the teardrop on the ipsilateral side beyond the healthy side. **(B)** The anteroposterior pelvic radiograph of the patient was placed on a standard grid line, and the horizontal distance of the femoral head rotational center to the ipsilateral teardrop was measured bilaterally. The yellow lines indicate the distance of the femoral head rotational center on the healthy side from the ipsilateral tear drop in the horizontal direction, and the blue lines indicate the portion of the femoral head rotational center on the affected side from the horizontal distance from the teardrop on the ipsilateral side relative to the healthy side beyond. **(C)** In patients with long-term unreduced posterior hip dislocation, the femoral head on the affected side detaches from the original acetabulum, and a hypodense ovoid area, known as the image of the real acetabulum, can be seen in the position of the original acetabulum. **(D)** Quasi-circular high-density area can be found in the anteroposterior X-ray examination, similar to the lunar aureole (pseudoacetabulum) around the moon (femoral head) wrapped around the femoral head, and is called the “aureole sign.”

In the first set of experiments, four signs were selected in the anteroposterior radiograph of the pelvis (Figure 1). (1) Rotational center shift upward: The rotational center of the femoral head was determined bilaterally, and the relative position (vertical distance) between the bilateral rotational center and the teardrop was measured, comparing the healthy side with the affected side. If the relative position (vertical distance) between the rotational center of the femoral head and the teardrop on the affected side was greater than that on the healthy side, it was defined as an upward shift of the rotational center (10, 11). (2) Rotational center shift outward: The relative position (horizontal distance) between the bilateral rotational center and the teardrop was measured, and the healthy side was compared with the affected side. If the relative position (horizontal distance) between the affected femoral head rotational center and the teardrop was greater than that of the healthy side, an external displacement of the rotational center was defined. (3) Image of real acetabulum: Real acetabulum refers to the original, undamaged acetabulum in cases of hip dislocation, where the femoral head is no longer located within the acetabulum. On the X-ray of the long-term unreduced posterior hip distribution, it can be observed that a slightly low-density oval area appears on the original acetabulum, and the center of this area is close to the center of the semicircle of the acetabulum on the healthy side and the relative position of the teardrop. (4) Aureole sign: A quasi-circular high-density area can be found in the anteroposterior X-ray examination, similar to the lunar aureole (pseudoacetabulum) around the moon (femoral head) wrapped around the femoral head, and is called the “aureole sign” (7). The experimental group in which the diagnostic value of these signs was assessed was divided into two parts: independent and combined indicators. Four signs as four independent signs formed the first four indicators of the first experimental group, two signs of superior and external rotational center were combined as the fifth indicator to diagnose the disease, and four imaging signs were combined as the sixth indicator to diagnose the disease (only when these four signs were observed simultaneously was the diagnosis of long-term unreduced posterior hip dislocation considered to be confirmed on the anteroposterior radiograph of the pelvis).

**Figure 2:**
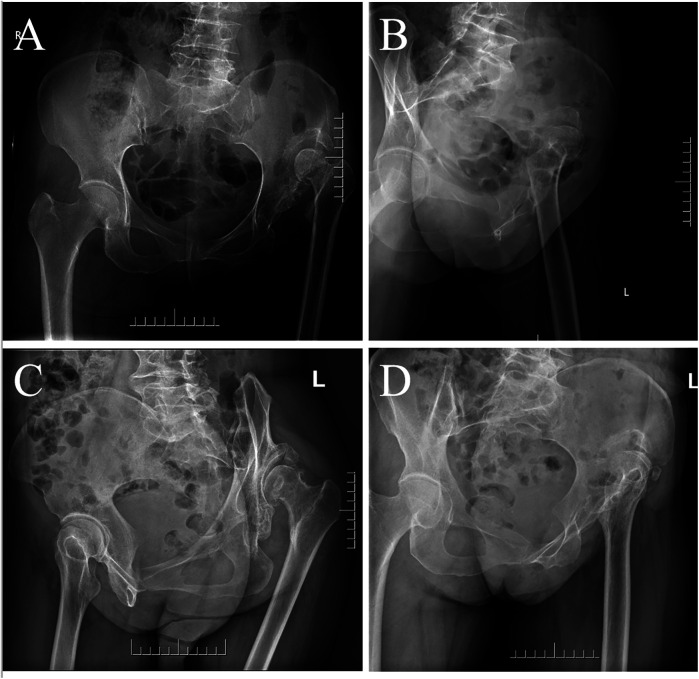
Long-term unreduced posterior hip dislocation in different X-ray projection postures. **(A)** Anteroposterior radiograph of the pelvis. **(B)** Lateral radiograph of the femur. **(C)** Obturator oblique radiograph of the pelvis. **(D)** Ilium oblique radiograph of the pelvis.

The second set of experiments covered a total of four X-ray projection positions, including one anterior–posterior and three lateral positions (Figure 2). (1) The anteroposterior radiograph of the pelvis was obtained with the patient in the supine position, with both lower extremities straightened, the feet tilted slightly inward, the toes close together, and bilateral anterior superior iliac spines equidistant from the table. The X-ray was vertically shot centered on the midpoint of the line between the anterior superior iliac spine and the upper edge of the pubic symphysis. (2) The lateral radiograph of the femur was obtained with the patient in the lateral decubitus position, with the examined side close to the table, the examined hip extended, and the sagittal plane of the femur parallel to the bed surface. The X-ray was vertically shot centered on the upper femur. (3) The obturator oblique radiograph of the pelvis was obtained with the patient in the supine position, the hip of the examined side was elevated so that the coronal surface of the body was at 45° to the bed surface, and the X-ray was vertically shot centered on the hip joint of the examined side. (4) The ilium oblique radiograph of the pelvis was obtained with the patient in the supine position, the contralateral hip was elevated so that the coronal surface of the body was at 45° to the bed surface, and the X-ray was vertically shot centered on the hip joint of the examined side. Images of these projected body positions were available for both the dislocation and control groups selected for this experiment. On the anteroposterior pelvic radiographs, the four signs mentioned in the first set of experiments were observed simultaneously before being judged as positive. On the three lateral images, images in which both upward and backward shifts of the rotational center were observed were considered positive images.

**Figure 3:**
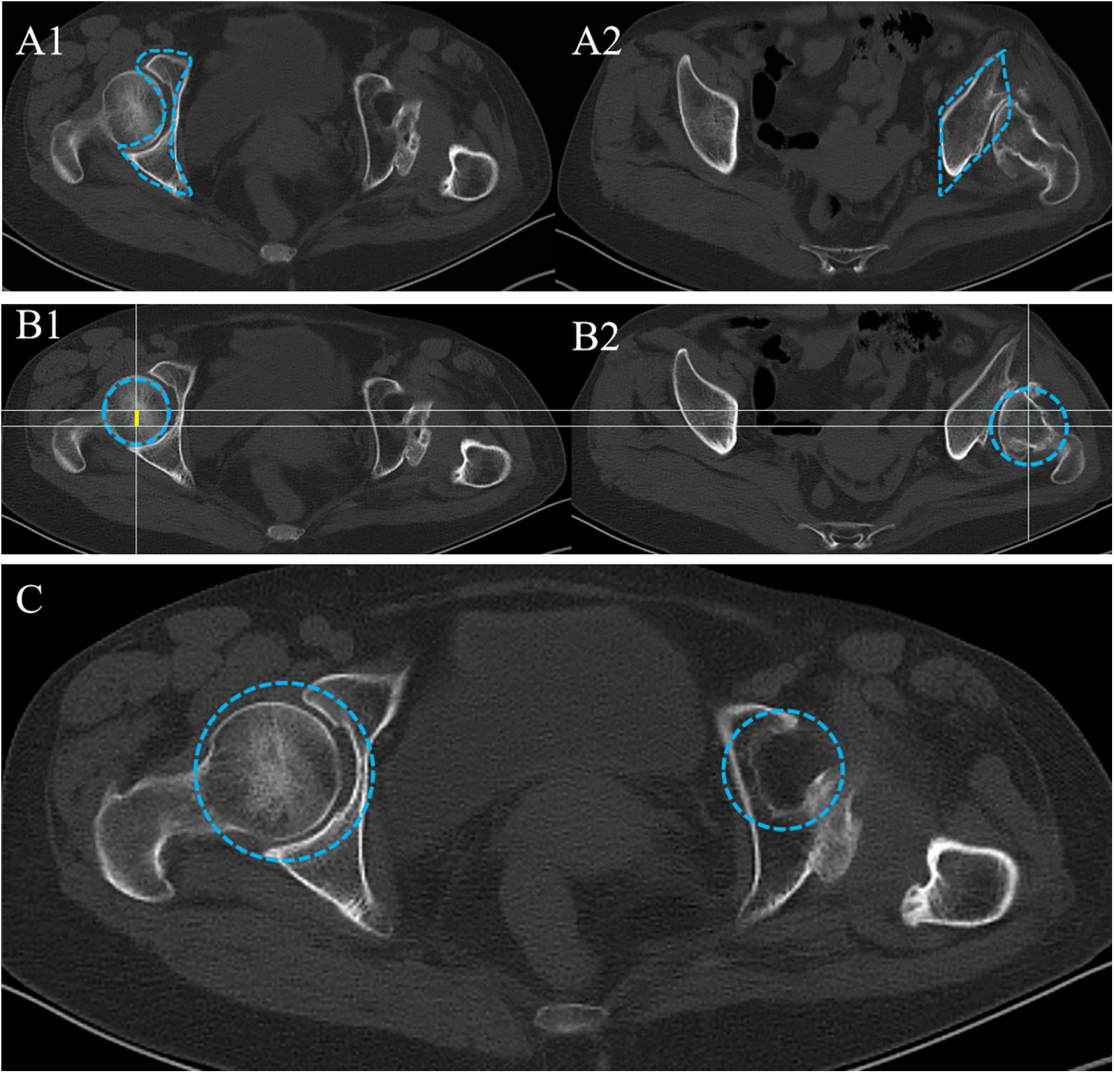
Axial CT radiological characteristics in patients with long-term unreduced hip joint dislocation. **(A1)** On the healthy side, the circumference of the femoral head on axial CT consists of the anterior, medial, and posterior columns of the acetabulum together into a region resembling a capital letter “I.” **(A2)** On the affected side, the area immediately adjacent to the femoral head is the ilium, which consists of the ilium, the false acetabulum, and the posterior wall of the original acetabulum around the posteriorly dislocated femoral head in an approximately rhomboid-like area called the rhomboid sign. **(B)** The patient's axial CT was placed in a standard grid line. The circular area enclosed by the blue dotted line was the affected side femoral head **(B1)** and the healthy side femoral head **(B2)**. The yellow line indicates the distance of the rotation center of the affected side femoral head relative to the healthy side posteriorly. **(C)** The position of the ipsilateral original acetabulum allows a semicircular area, which is mirrored with the healthy acetabulum in a relatively small to healthy ratio, which is an image of an atrophic acetabulum on the affected side.

The third part of the study was to observe the axial CT images of the hip joint with three signs (Figure 3). (1) Rhombus sign: In healthy people, the anterior column, inner wall, and posterior column of the acetabulum resemble a capital letter “I.” However, in the case of long-term unreduced posterior hip dislocation, the ilium, dome of the original acetabulum, and internal or posterior wall of the pseudoacetabulum form a rhombus (7). (2) Rotational center shift rearward: Comparison with the healthy side on axial CT shows that the rotational center of the femoral head on the affected side has shifted posteriorly. (3) Atrophic true acetabulum: When the femoral head has been divorced from the acetabulum for a long time, the original acetabulum shrinks gradually over time, and due to atrophy, the acetabulum loses its normal half-enclosed shape.

**Figure 4:**
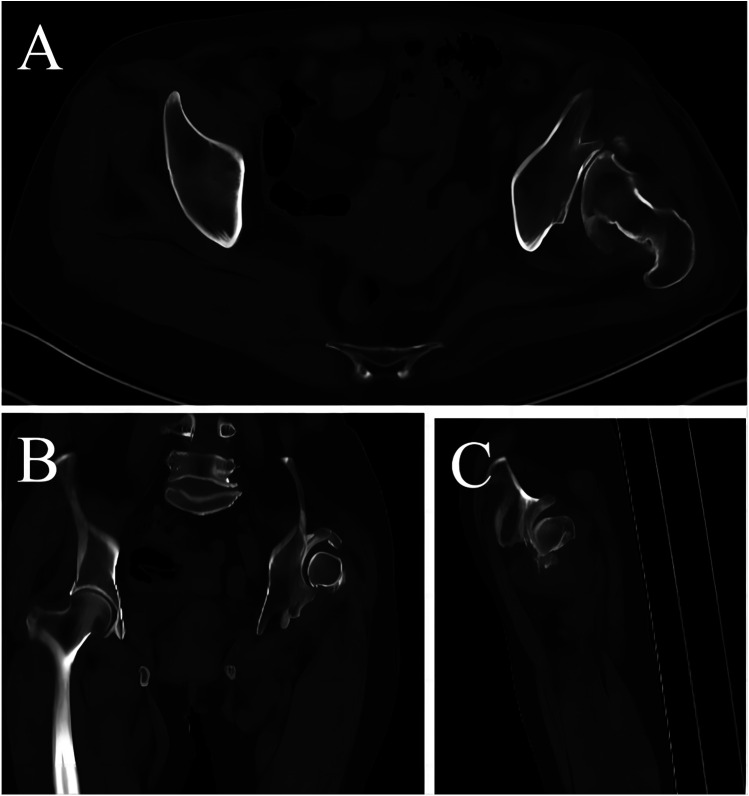
Different types of CT reformation techniques for long-term unreduced posterior hip dislocation. **(A)** Axial CT. **(B)** Coronal multiplanar reformation. **(C)** Sagittal multiplanar reformation.

The fourth part of the study covered the analysis of three types of images: axial CT, coronal reconstruction, and sagittal reconstruction (Figure 4). Axial CT was obtained by helix CT scan, and the slice thickness selected for both CT reconstructions was 5 mm. The observed indexes for all three CT images were the posterior and superior shift of the rotational center. Additionally, added was a combined index, that is, the aureole signs on anteroposterior radiographs of pelvic and rhombus signs on axial CT.

Two orthopedists with >10 years of practice were included in this trial. They were not involved in the diagnosis or treatment of the patients in the dislocation group. The same experienced radiologist trained both orthopedists in each group, and both orthopedists were involved in each review. In the same set of experiments, both orthopedists were given the same observation index and judged 300 images separately, and the results of both orthopedists' judgments were recorded. The anatomical results seen during surgery (discharge diagnosis) were used as the gold standard (true positive).

The results obtained will be compared with the gold standard, and each set of results will be recorded in a four-compartment table, with sensitivity, specificity, accuracy, Youden's index, positive predictive value (PPV), negative predictive value (NPV), and interobserver agreement calculated and recorded. After completion of the five sets of experiments, all experiments will be repeated and compared with the results of the initial experiment, and intraobserver consistency will be recorded. With sensitivity as the primary indicator and other indicators as secondary indicators, the data obtained will be used to perform statistical analysis to determine which signs and shooting angles are valuable for the diagnosis of this disease. The obtained results were contrasted with the blank group, the chi-square test was performed, and the *P*-value was recorded.

All statistical analyses were performed with SPSS version 22.0 (SPSS Inc., Chicago, IL, USA). All data were collected in Excel, and a database was established to facilitate statistical analysis. We calculated the sensitivity, specificity, accuracy, Youden's index, positive predictive value (PPV), and negative predictive value (NPV). Youden's index, which is calculated as sensitivity + specificity − 1, was used to evaluate the overall performance of the test. We used the intraclass correlation coefficient (ICC) to determine intra- and interobserver reliability for every measurement and ratio. Intraobserver consistency refers to the degree of agreement when the same observer repeats the measurements, while interobserver consistency refers to the degree of agreement between different observers. An ICC > 0.80 was considered excellent.

## Case presentation

To further illustrate the key diagnostic imaging features of long-term unreduced posterior hip dislocation, we present two representative cases.

### Case 1: a 55-year-old male patient

A 55-year-old male patient (Table 1, Case 7) presented in April 2020 with left hip pain and restricted mobility (Figure 1).

**Table 1:** Demographics and general characteristics of patients with long-term unreduced posterior hip dislocation.

Case	Sex	Age (years)	Side	Etiology	Comorbidity injuries	Time from initial injury to surgery (years)	Preoperative Harris score	Scoliosis	Adduction, internal rotation (affected side)
1	Male	38	Left	Fall from a height	–	6	35	+	+
2	Female	39	Left	Traffic accident	Acetabular fracture	9	36	+	+
3	Male	59	Left	Traffic accident	Patella fracture, tibiofibular fracture, iliac fracture	9	38	+	+
4	Female	46	Right	Traffic accident	Acetabular fracture	9	42	+	+
5	Female	76	Right	Traffic accident	Acetabular fracture	5	42	+	+
6	Male	63	Right	Traffic accident	–	19	42	+	+
7	Male	55	Left	Traffic accident	Acetabular fracture	11	43	+	+
8	Male	70	Left	Traffic accident	Acetabular fracture	12	43	+	+
9	Male	65	Right	Crushing injury	–	7	43	+	+
10	Female	71	Right	Traffic accident	Femoral shaft fracture	6	44	+	+
11	Female	73	Right	Traffic accident	–	20	44	+	+
12	Female	71	Right	Fall from a height	Femoral shaft fracture	15	44	+	+
13	Female	56	Right	Crushing injury	Acetabular fracture	8	45	+	+
14	Female	56	Left	Crushing injury	Superior pubic ramus fracture	23	45	+	+
15	Male	62	Right	Fall from a height	Calcaneal fracture	6	46	+	+
16	Female	52	Left	Fall from a height	Acetabular fracture	3	46	+	+
17	Male	75	Left	Traffic accident	Acetabular fracture	17	46	+	+
18	Female	69	Right	Fall from a height	Acetabular fracture	8	48	+	+
19	Male	59	Left	Fall from a height	Ischial fracture	8	48	+	+
20	Male	62	Left	Traffic accident	–	12	49	+	+
21	Male	67	Left	Traffic accident	–	7	49	+	+
22	Male	42	Right	Traffic accident	Inferior pubic ramus fracture	13	51	+	+
23	Female	59	Left	Traffic accident	–	8	51	+	+
24	Male	58	Left	Crushing injury	Calcaneal fracture	14	52	+	+

The patient had sustained a left hip injury 11 years prior due to a traffic accident but did not receive appropriate treatment.

On physical examination, he exhibited a limping gait, left lower limb shortening of approximately 1 cm, and severely restricted hip range of motion.

The anteroposterior pelvic radiograph (Figure 1) demonstrated an aureole sign, a high-density ring-like structure surrounding the dislocated femoral head. Additional findings included superior and lateral displacement of the femoral head and the presence of a pseudoacetabulum.

The patient was diagnosed with long-term unreduced posterior hip dislocation and underwent THA. Postoperatively, pain relief and functional improvement were achieved.

### Case 2: a 59-year-old female patient

A 59-year-old female patient (Table 1, Case 23) presented in August 2021 with left hip pain and gait abnormalities (Figures 2–4).

The patient had sustained a left hip injury eight years prior due to an accident but did not seek medical attention immediately due to financial constraints.

On physical examination, she ambulated with a cane and exhibited severe scoliosis, left lower limb shortening of approximately 4 cm, and severely restricted hip range of motion.

The anteroposterior pelvic radiograph (Figure 2A) showed an aureole sign, femoral head rotational center displacement, and a pseudoacetabulum. The lateral (Figure 2B), obturator oblique (Figure 2C), and ilium oblique radiographs (Figure 2D) revealed significant posterior and superior displacement of the femoral head.

Axial CT (Figure 3A2) demonstrated a rhombus sign, with additional findings of posterior displacement of the femoral head rotational center (Figure 3B) and an atrophic true acetabulum (Figure 3C).

Multiplanar CT reconstructions (Figures 4A–C) further delineated the extent of acetabular remodeling and femoral head displacement.

The patient was diagnosed with long-term unreduced posterior hip dislocation and underwent THA. To prevent excessive sciatic nerve traction, full restoration of limb length was not performed, leaving a postoperative limb length discrepancy of approximately 1 cm. The patient achieved pain relief and improved hip mobility.

## Results

Table 2 summarizes the diagnostic value of various X-ray signs for identifying long-term unreduced posterior hip dislocation. For the blank group (X-ray), the diagnostic metrics were as follows: sensitivity = 25.00%, specificity = 92.03%, accuracy = 86.67%, Youden's index = 0.170, PPV = 21.43%, NPV = 93.38%, intraobserver consistency = 0.963, and interobserver consistency = 0.950. The observer reliability of all indicators was above 0.800. For the main observation of this study (sensitivity), the highest sensitivity of 62.50% was obtained for the rotational center shift upward + outward. The data for the rotational center shift upward and the aureole sign were in the second and third positions with 58.33% and 45.83%, respectively. The combined index of the four signs (41.67%) and the image of the real acetabulum (37.50%) were the fourth and fifth, respectively. The rotational center shift outward showed a low sensitivity of 29.17% when used as an independent indicator. In terms of specificity, the combined index of the four signs had a specificity of 91.30%. The specificity of the two observed indicators of rotational center shift upward and rotational center shift outward was the next highest, 88.41%. The specificity of the aureole sign was 81.52%. The specificity of the rotational center shift upward did not reach 80%. In terms of accuracy, the top three indexes were the combined four-sign indicator (87.33%), the two observed indicators of rotational center shift upward and rotational center shift outward (86.33%), and the image of the real acetabulum (81.33%). In terms of Youden's index, the rotational center shift upward + outward was the highest at 0.509. Youden's index of the rotational center shift upward, the four signs joint index, and the aureole sign were in the range of 0.226–0.355. The rotational center shift outward was the lowest, at 0.121. The PPV of all indicators was below 35% (12.96%–31.91%). The NPV of all six indicators was above 90% (93.09%–96.44%).

**Table 2:** Comparison of the diagnostic value of different X-ray signs on anteroposterior radiographs of the pelvis for diagnosing long-term unreduced posterior hip dislocation.

Signs & *P*-values	Sensitivity	Specificity	Accuracy	Youden's index	PPV	NPV	Intraobserver consistency	Interobserver consistency
Blank group (X-ray)	12.50%	89.13%	83.00%	0.016	9.09%	92.13%		
Aureole sign	45.83%	81.52%	78.67%	0.274	17.74%	94.54%	0.930	0.903
*P*	0.024	0.012	0.178		0.407	0.282		
Rotational center shift upward	58.33%	77.17%	75.67%	0.355	18.18%	95.52%	0.863	0.843
*P*	<0.001	<0.001	0.027		0.227	0.126		
Rotational center shift outward	29.17%	82.97%	78.67%	0.121	12.96%	93.09%	0.883	0.847
*P*	0.155	0.037	0.178		0.583	0.680		
Image of the real acetabulum	37.50%	85.14%	81.33%	0.226	18.00%	94.00%	0.883	0.870
*P*	0.046	0.162	0.594		0.259	0.405		
Rotational center shift upward + outward	62.50%	88.41%	86.33%	0.509	31.91%	96.44%	0.870	0.877
*P*	<0.001	0.787	0.257		0.016	0.035		
Rotational center shift upward + outward + image of the real acetabulum + “aureole” sign	41.67%	91.30%	87.33%	0.330	29.41%	94.74%	0.960	0.947
*P*	0.023	0.390	0.135		0.035	0.225		

Table 3 summarizes the diagnostic value of different X-ray projection postures for identifying long-term unreduced posterior hip dislocation. Intraobserver consistency and interobserver consistency of 0.800 or more. In terms of sensitivity, the highest one was the obturator oblique radiograph of the pelvis (sensitivity = 58.33%, specificity = 82.25%, accuracy = 80.33%, Youden's index = 0.406). The sensitivity of lateral radiographs of the femur and ilium oblique radiographs of the pelvis was <30%. The highest specificity, accuracy, and PPV were found for the anteroposterior pelvic radiographs. The highest Youden's index was found for the obturator oblique radiograph of the pelvis. The NPV of these four projection positions of the reading results did not differ significantly (92.41%–95.78%).

**Table 3:** Comparison of the diagnostic value of different X-ray projection postures for diagnosing long-term unreduced posterior hip dislocation.

Signs & *P*-values	Sensitivity	Specificity	Accuracy	Youden's index	PPV	NPV	Intraobserver consistency	Interobserver consistency
Blank group (X-ray)	12.50%	89.13%	83.00%	0.016	9.09%	92.13%		
Anteroposterior radiograph of the pelvis	45.83%	88.41%	85.00%	0.342	25.58%	94.94%	0.933	0.917
*P*	0.011	0.787	0.504		0.066	0.192		
Lateral radiograph of the femur	25.00%	79.35%	75.00%	0.044	9.52%	92.41%	0.943	0.780
*P*	0.267	0.002	0.016		0.945	0.910		
Obturator oblique radiograph of the pelvis	58.33%	82.25%	80.33%	0.406	22.22%	95.78%	0.923	0.900
*P*	0.001	0.021	0.399		0.109	0.089		
Ilium oblique radiograph of the pelvis	29.17%	81.16%	77.00%	0.103	11.86%	92.95%	0.890	0.827
*P*	0.155	0.008	0.066		0.682	0.729		

For the blank group (CT), the diagnostic metrics were as follows: sensitivity = 25.00%, specificity = 92.03%, accuracy = 86.67%, Youden's index = 0.170, PPV = 21.43%, NPV = 93.38%, intraobserver consistency = 0.963, and interobserver consistency = 0.950. Table 4 summarizes the diagnostic value of axial CT signs in identifying long-term unreduced posterior hip dislocation. Three independent indicators can be found by observing the patient's image on CT, namely, rhombus sign, rotational center rearward shift, and atrophic real acetabulum. First, for the rhombus sign, sensitivity = 70.83%, specificity = 90.94%, accuracy = 89.33%, Youden's index = 0.618, PPV = 40.48%, NPV = 97.29%, intraobserver consistency = 0.943, and interobserver consistency = 90.00%. Second, for the rotational center shift rearward, sensitivity = 62.50%, specificity = 88.77%, accuracy = 86.67%, Youden's index = 0.513, PPV = 32.61%, NPV = 96.46%, intraobserver consistency = 0.933, and interobserver consistency = 0.870. Third, for atrophic true acetabulum, sensitivity = 54.17%, specificity = 88.41%, accuracy = 85.67%, Youden's index = 0.426, PPV = 28.89%, NPV = 95.69%, intraobserver consistency = 0.920, and interobserver consistency = 0.910.

**Table 4:** Comparison of the diagnostic value of axial CT signs for diagnosing long-term unreduced posterior hip dislocation.

Signs & *P*-values	Sensitivity	Specificity	Accuracy	Youden's index	PPV	NPV	Intraobserver consistency	Interobserver consistency
Blank group (CT)	25.00%	92.03%	86.67%	0.170	21.43%	93.38%		
Rhombus sign	70.83%	90.94%	89.33%	0.618	40.48%	97.29%	0.943	0.900
*P*	0.001	0.511	0.049		0.026	0.029		
Rotational center shift rearward	62.50%	88.77%	86.67%	0.513	32.61%	96.46%	0.933	0.870
*P*	0.019	0.118	0.723		0.428	0.174		
Atrophic true acetabulum	54.17%	88.41%	85.67%	0.426	28.89%	95.69%	0.920	0.910
*P*	0.009	0.015	0.301		0.563	0.133		

Table 5 records the observation results of three types of CT and the aureole sign + rhombus sign for long-term unreduced posterior hip dislocation. Interobserver consistency and interobserver consistency for three types of CT and the aureole sign + rhombus sign was above 0.850. The sensitivities of the three types of CT were 70.83%, 58.33%, and 54.17%, respectively. The specificity was higher than 87%, and the accuracy was above 85% for all of them. The highest Youden's index was for axial CT, with 0.640. Youden's index of coronal multiplanar reformation was in the middle at 0.478, and sagittal multiplanar reformation was the lowest at 0.419. The highest data for all indicators were obtained when the aureole sign + rhombus sign was used as a combined indicator, sensitivity = 79.17%, specificity = 97.10%, accuracy = 95.67%, Youden's index = 0.763, PPV = 70.37%, NPV = 98.17%, intraobserver consistency = 0.960, and interobserver consistency = 0.946.

**Table 5:** Comparison of the diagnostic value of different types of CT reformation techniques and the aureole + rhombus signs for diagnosing long-term unreduced posterior hip dislocation.

Signs & *P*-values	Sensitivity	Specificity	Accuracy	Youden's index	PPV	NPV	Intraobserver consistency	Interobserver consistency
Blank group (CT)	25.00%	92.03%	86.67%	0.170	21.43%	93.38%		
Axial CT	70.83%	93.12%	91.33%	0.640	47.22%	97.35%	0.900	0.910
*P*	0.001	0.626	0.068		0.033	0.029		
Coronal multiplanar reformation	58.33%	89.49%	87.00%	0.478	32.56%	96.11%	0.913	0.887
*P*	0.019	0.304	0.904		0.308	0.162		
Sagittal multiplanar reformation	54.17%	87.68%	85.00%	0.419	27.66%	95.65%	0.907	0.913
*P*	0.039	0.091	0.558		0.578	0.255		
Aureole sign + rhombus sign	79.17%	97.10%	95.67%	0.763	70.37%	98.17%	0.960	0.947
*P*	<0.001	0.009	<0.001		<0.001	0.005		

## Discussion

**Figure 5:**
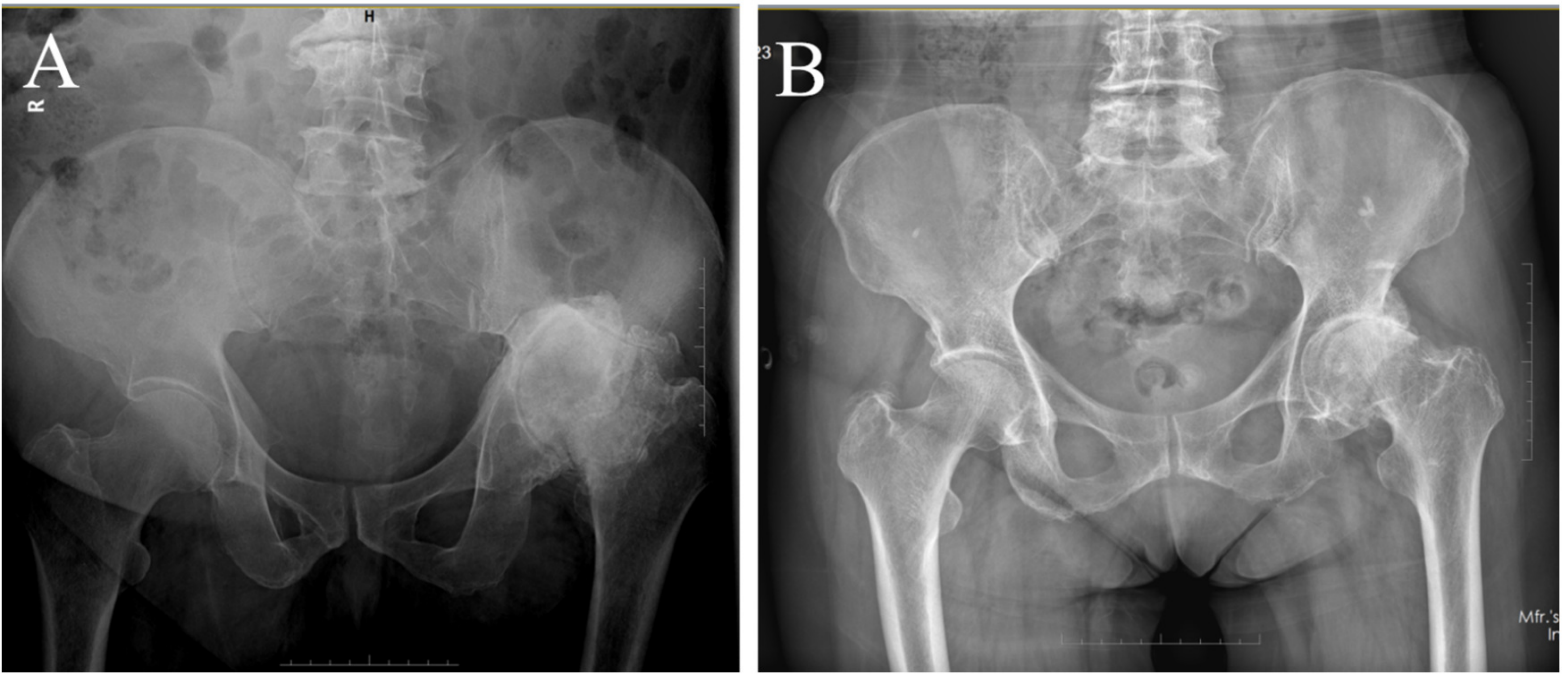
Long-term unreduced hip joint dislocation, arthritis of the hip on the anteroposterior radiograph of the pelvis. **(A)** Rotational center shift upward and outward, image of the real acetabulum and aureole sign can be observed simultaneously on images of long-term unreduced hip joint dislocation. **(B)** On images of osteoarthritis of the hip, there is no upward migration or exteriorization of the center of rotation.

In that study, the duration of illness at inclusion in the dislocation group was >1 year. Pai (12), Nagi et al. (9), Ilyas and Rabbani (8), and we all found that pseudoacetabulum formation after hip dislocation mostly appeared in patients who had been injured for >1 year without effective treatment and was often misdiagnosed as other end-stage hip diseases, such as OA of the hip and osteonecrosis of the femoral head. OA of the hip leads to joint pain, stiffness, and decreased range of motion (13–17). A study in Burgess found that adults with moderate to severe hip osteoarthritis can have deficits in strength or endurance (18), pain symptoms may appear more frequently later in the course of the disease (19), and patients with long-term unreduced posterior hip dislocation similarly present because of hip pain as well as limited mobility. OA causes joint space narrowing, osteophyte formation, and sclerosis (20, 21), with similar radiographic features to those seen in long-term unreduced posterior hip dislocation. Therefore, we included OA of the hip as a control group in this study (Figure 5). The radiographic examinations chosen for this study were X-ray and CT, and X-ray images are the most easily performed tests for evaluating hip disease (21). CT can overcome some of the limitations of X-ray examination and can more precisely assess the anatomical relationship, location, and morphology of the various constituent bone structures that make up the hip joint (22). Owing to MRI having no more advantages for imaging bone than the first two, the study did not incorporate routine examination applications.

Patients in the dislocation group had a history of high-energy trauma, such as traffic accidents and crush injuries, and 70.83% of them had comorbidities, including multiple fractures and acetabular fractures. These patients had posterior hip dislocation that was not promptly detected or treated incorrectly for several reasons, and the acetabular dislocation was not corrected. Only one patient in the control group had previously undergone reduction for a posterior hip dislocation. However, its restoration was not successful, or the dislocation occurred again within a short time after restoration and was not corrected twice. Thompson and Epstein (23) reported that recurrent hip dislocation was very rare, occurring in only 1.5% of 204 patients. We recorded some of the patients' postural features, and all had scoliosis of varying degrees with adduction, internal rotation, flexion, and shortening deformities of the affected limbs. The sensitivity of the blank group (X-ray) and blank group (CT) was 12.50% and 25.00%, respectively, which was relatively consistent with clinical practice.

### Diagnostic value of multiangle radiographic imaging

The purpose of the study was to improve the accuracy of the diagnosis of long-term unreduced posterior hip dislocation by means of multiangle orientation X-rays. In this study, the anteroposterior radiographs of the pelvis focused on four separate indices: (1) rotational center shift upward, (2) rotational center shift outward, (3) real acetabulum location and radiographic characteristics, and (4) mechanism and incidence of the aureole sign.

The reason why displacement of the rotational center of the femoral head on the affected side occurs is related to the anatomic shape of the pelvis, and a posteriorly dislocated femoral head will undergo upward outward displacement due to the surrounding bony blockade. An image of the real acetabulum was generated because the femoral head that should be wrapped by the acetabulum is not in the original position, thus making no occlusion of the femoral head. The projection of the acetabulum alone appears on the image taken anteroposterior to the pelvis. We did not find definitive literature on the mechanism of pseudoacetabulum formation. We hypothesize that the appearance of the aureole sign is related to the formation of a pseudoacetabulum, which is essentially an area of high density in the anterior–posterior position of the pelvis. The pathological change of pseudoacetabulum was mentioned in other scholars' studies. According to the description of the Hartofilakidis et al. (24) classification system for congenital hip disease in adults, high dislocation of the hip is divided into two subtypes, C1 (the femoral head articulates with a false acetabulum) and C2 (no false acetabulum; the femoral head is free-floating within the gluteal musculature). In the published literature, adequate intra- and interobserver reliability was reported for Hartofilakidis et al. (25). However, the mechanism of pseudoacetabulum formation was not mentioned in their study either.

Based on the statistical results, it can be seen that when these four indicators appear independently as diagnostic points, they have a high observer agreement. The rotational center shift upward, the image of the real acetabulum, and the aureole sign show high sensitivity, specificity, and accuracy and have good value. However, when the rotational center shift outward as an independent indicator, both sensitivity (*P* = 0.155) and positive predictive values were low, with a Youden's index of only 0.121. The statistical results showed that there was no statistical significance when solely the rotational center shift outward was taken as an independent observation. Therefore, we took the displacement of the rotational center (including shifting upward and shifting outward) as the fifth diagnostic indicator. In the end, four signs were told to the observers at the same time, and the diagnosis was made only when the observers met the four signs at the same time. Analysis of the data showed that the most valuable sign on images of the anteroposterior radiograph of the pelvis was the rotational center shift upward. In posterior dislocation of the hip, the femoral head on the affected side is displaced upwards due to the morphology of the pelvis itself and the action of the lower limb muscles, similar to developmental dysplasia of the hip (DDH). It is difficult to observe that this condition rarely occurs in hip OA included in the control group, so the sign has a high sensitivity in this study, and the identification between long-term unreduced posterior hip dislocation and DDH was not included in this trial. However, there is still a non-negligible risk of misdiagnosis because there are incomplete and complete dislocations of long-term unreduced posterior hip dislocation, and in the case of incomplete dislocations the low-level dislocation may be overlooked due to interference from the bone.

The aureole sign is a valuable sign of long-term unreduced posterior hip dislocation. However, in the present study, the aureole sign was not the most valuable sign on the anteroposterior radiograph of the pelvis, and this finding was associated with imaging changes in OA.OA has now been well studied and causes narrowing of the joint space, formation of bone fragments, and sclerosis in the affected joints (21, 22). Assessment of osteophytes, joint space narrowing, subchondral sclerosis, and femoral head and acetabular deformities are also signs used by the Kellgren and Lawrence system (K & L), the most popular diagnostic modality for OA. Other imaging classifications such as Croft's grade (26) and Tönnis classification also exist (27). Some radiographic features of hip OA are described in the radiographic atlas of osteoarthritis developed by Altman and Gold (21), with more sites of possible osteophyte formation, including the superior and inferior acetabular margins and the superior and inferior segments of the femoral head. Osteophyte formation at multiple sites or even at all sites may occur simultaneously. We considered that the control OA patients had some areas with larger areas of osteophytes that formed large areas of hyperdensity on the anteroposterior radiograph of the pelvis images, which created some areas of hyperdensity that were larger but irregularly shaped. Whereas the dislocation group included patients with long-term unreduced posterior hip dislocation had a wide variation in the duration of illness, and there was a non-negligible difference in the degree of hyperostosis of the pseudoacetabulum, some cases of incomplete dislocation had an incomplete formation of the pseudoacetabulum, resulting in a semicircular shape or other irregular morphology, which was difficult to distinguish from the osteophytes of hip OA. As an independent diagnostic indicator, the specificity of the image of the real acetabulum is slightly higher, but other data are not outstanding, which may be because although this sign is indeed of high value, it is difficult to observe the image of the real acetabulum on anteroposterior radiographs of the pelvis due to reasons such as atrophy of the original acetabulum, and even the bone density in this area is increased due to reasons such as hyperosteogeny behind the original acetabulum. This makes it more difficult to distinguish the image of the real acetabulum that is not obvious because of the overlap of the front and rear images. There is another unexpected result. The experiment began to predict the diagnostic value of the rotational center shift outward higher, but it did not obtain an ideal result. This may be related to the low degree of shifting outward, and the patient's hip joint movement was limited, which made the body position unable to meet the standard during X-ray photography. Therefore, we took the displacement of the rotational center (including shifting upward and shifting outward) as a diagnostic indicator, and the results were satisfactory. Its sensitivity and specificity were more satisfactory than when they were used as independent indicators. The accuracy, positive predictive value, and negative predictive value were also improved to some extent. In the end, four signs were told to the observers at the same time, and the diagnosis was made only when the observers met the four signs at the same time. The specificity of the diagnosis was as high as 91.30%, while the sensitivity decreased significantly to only 41.67%. This finding does not indicate that not all signs appear simultaneously on an anteroposterior radiograph of the patient's pelvis, but that not every sign can present with typical radiographic features and therefore be not acutely detected by an orthopedist.

There are multiple projection postures to choose from when taking X-rays, and improving the reasonable examination of different projection postures can help to diagnose the disease. In the current diagnosis and treatment system, when encountering a patient with chronic hip joint activity limitation and pain, the anteroposterior radiograph of the pelvis is a routine examination item. However, for patients with hip joint posterior dislocation, can X-rays from other angles also help patients diagnose this disease? We selected four different projection postures in the second set of experiments: (1) anteroposterior radiograph of the pelvis, (2) lateral radiograph of the femur, (3) obturator oblique radiograph of the pelvis, and (4) ilium oblique radiograph of the pelvis. The results of the observer consistency test of the four indicators show that the reliability and stability of the observation results are good. On the anteroposterior radiograph of the pelvis, we informed the observers of four independent indicators (the same as in 1), and the results were consistent with Table 1. Three angles, namely, the lateral radiograph of the femur, observer object radiograph of the pelvis, and ilium objective radiograph of the pelvis, are all lateral images of the hip joint. On the X-ray images of the three types of laterals, high-density areas with different degrees of encapsulation in the femoral head of the affected side can be similarly seen. This is the radiographic appearance of a pseudoacetabulum on a lateral X-ray. Analysis of the extent of the hyperdense zone on each patient's anteroposterior radiographic image vs. his own lateral radiographic examination reveals essentially the same relative position of the pseudoacetabulum to the femoral head on the affected side in different patients, which is also consistent with the situation of pseudoacetabulum formation. Therefore, the indicators observed by the lateral observer include backward and upward movement of the center of rotation and imaging of the pseudoacetabulum in the lateral position. However, it cannot be accurately distinguished on a considerable part of the images because of interference from the image of the iliac bone on the lateral view. The specificity on X-ray of the three lateral positions is high, but the sensitivity of lateral radiograph of the femur and ilium oblique radiograph of the pelvis is low, 25.00% (*P* = 0.267) and 29.17% (*P* = 0.155), respectively. This has been expected by the experimental designer when capturing images. During the shooting, due to the limited hip joint activity of the patient, it was difficult for doctors to put the patient's position in a standard position. The patient was always in an inclined position during the shooting. When the captured image comes out, the inexperienced observers without information may not be able to discover that the image is tilted. Whereas the upward and outward displacements that occur with long-term unreduced posterior hip dislocation of an incompletely dislocated hip on a lateral X-ray are not very distinct, the displacement to the rotational center of the femoral head cannot be accurately identified. Surprisingly, except for the positive predictive value, all the other indicators on the obturator oblique radiograph of the pelvis were satisfactory. The displacement of the affected rotational center is more apparent in the obturator oblique radiograph of the pelvis views than in the other two lateral views, making it easier for the orthopedist to distinguish these signs.

CT can overcome some of the limitations of X-ray and allows a very accurate assessment of the anatomical relationships and morphology of the skeletal structures that make up the hip joint (22). In the study for the diagnosis of 101 consecutive acetabular fractures by Ohashi et al. (28), when using radiographs including Judet views, the interobserver agreement had a kappa value of 0.42, and when adding the MDCT with 2D MPR and 3D images, the interobserver agreement significantly improved to a kappa value of 0.70. This result was also confirmed in the research of Geijer and El-Khoury (29). Long-term unreduced posterior hip dislocation occurs after having experienced high-energy violent trauma, resulting in fractures such as those at the acetabular rim, with the femoral head dislocated in situ, a process that inevitably produces bone fragments around the femoral head. These bone fragments are not sensitive to imaging on X-rays, whereas CT is more sensitive than X-rays in detecting microsclerosis, subchondral cysts, small osteophytes, or epiphyses (30, 31). Therefore, CT may also provide aid in improving the diagnostic yield of long-term unreduced posterior hip dislocation.

### Diagnostic value of CT

Several independent indicators that may improve the diagnostic value of long-term unreduced posterior hip dislocation can also be found on CT. Axial CT is a kind of cross-sectional imaging. Compared with anteroposterior pelvic radiographs, axial CT images can provide a more intuitive and clear view of the rotational center of posterior hip dislocation. Three indicators were selected to help diagnose the disease. The observer consistency test of these indicators suggested that the three indicators had good reliability. The first is the rhombus sign, which as an indicator has a high sensitivity, specificity, and other datum. The second indicator is the rotational center rearward shift. Because the image of axial CT was a cross section, when the healthy side was used as a contrast, it was very obvious to see the rearward movement of the femoral head rotational center on the affected side. The rearward shift of the rotational center was not a specific indicator of this disease, although other situations (such as sudden high-energy violence leading to simultaneous rearward movement of the acetabulum and femoral head and malunion) are extremely rare. However, when shooting, the patient's position will also lead to a higher healthy side, and the horizontal direction has a certain clockwise or anticlockwise tilt angle. Therefore, as an independent diagnostic indicator, the rotational center rearward shift also has a high diagnostic value, although the positive predictive value is low. The third indicator is atrophic true acetabulum. Because the femoral head has been divorced from the acetabulum for a long time, the original acetabulum shrinks gradually over time, and because of atrophy, the shape of the acetabulum is no longer half-enclosed under normal conditions. Experienced doctors can observe this sign sensitively. We are not aware of relevant reports as to why atrophy of the original acetabulum occurs. Presented a conjecture in the study of Liu et al. (7), they speculated that this phenomenon might be related to the lack of noncontact stress stimulation between the original acetabulum and the femoral head. Alternatively, in some cases of combined acetabular fracture, “atrophy” is the appearance of the original acetabular bone defect. Our researchers maintain agreement with this assumption. When the atrophic true acetabulum was used as an independent observation measure, its sensitivity and specificity were lower than the other two measures. This result may result from two factors. First, because of the atrophy of the acetabulum and its severely distorted shape, combined with the incorrect position of the patient resulting in the existence of an oblique angle on cross section, the acetabular image truly located at the same horizontal plane cannot be clearly observed on axial CT. Second, acetabular hyperostosis is extremely common in images of control hips with OA (20, 21). Whereas an atrophic acetabulum on an image of a long-term unreduced posterior hip dislocation will show a similar picture of osteophyte formation, this can cause mistakes when reviewing the image, especially when faced with a long-term unreduced hip with an incomplete dislocation that is not apparent with increased spacing of the femoral head from the acetabulum.

In addition to axial CT, it can also be used for coronal multiplanar reformation and sagittal multiplanar reformation. During the coronal multiplanar reformation, the posterior dislocated femoral head would lead to the hip joints on both sides not appearing in the same coronal plane, even the coronal plane where the contralateral femoral head is located is relatively distant from that where the ipsilateral femoral head is located. It is better to be diagnosed on images, and the data results are relatively good. However, the results of sagittal multiplanar reformation did not meet expectations, especially the positive predictive value. After the lateral X-ray test, the experimenters believed that the posterior dislocated femoral head could be observed more clearly and intuitively during sagittal multiplanar reformation. However, from the experimental data results, although the sensitivity was higher than that of the lateral radiograph of the femur and ilium oblique radiograph of the pelvis, it did not exceed that of the obturator oblique radiograph of the pelvis. The specificity of sagittal multiplanar transformation was lower than that of lateral X-ray images, and the positive predictive value was also significantly lower than expected. The experimenters did not obtain a convincing result based on this result. For the combined index, including the aureole sign on the anteroposterior radiograph of pelvic and rhombus sign on axial CT, the sensitivity, specificity, and accuracy were 79.17%, 97.10%, and 95.67%, respectively. According to Liu et al. (7), in X-ray + CT + 3D reconstruction, the sensitivity, specificity, and accuracy were 93.8%, 99.6%, and 96.3%, respectively. As 3D reconstruction was not performed in our study, the conclusions can be considered compatible by comparison with the findings of these individuals.

Existing reports also mention the limitations of CT examination, including its limited spatial resolution and contrast resolution preventing reliable evaluation of the joint soft tissues (cartilage and labrum) (22). The femoral head or acetabulum in the case of long-term unreduced posterior hip dislocation is prone to cartilage fragment formation due to high-energy violent trauma (32), and soft tissue involvement, including acetabular labrum or capsular invagination, is the cause of repeated dislocation in young patients (33, 34). However, arthrography can provide better visualization in these cases (32), as radiological examinations are easy to perform, inexpensive, and non-invasive, making them indispensable tests (35).

Radiologists should incorporate the identified radiographic markers (e.g., rotational center shift, pseudoacetabulum, and aureole sign) in routine X-ray and CT assessments of patients with suspected long-term unreduced posterior hip dislocation. Orthopedic surgeons should use these markers to guide diagnosis and treatment, particularly for patients with a history of high-energy trauma and limited hip mobility. Using multiple imaging angles can improve diagnostic accuracy, helping to determine the need for surgical intervention.

Future research should validate these findings through prospective studies with larger, multicenter samples to enhance generalizability. Studies should also explore advanced imaging techniques such as MRI and 3D reconstructions to further refine diagnostic accuracy and assess the clinical impact of these radiographic markers on treatment outcomes.

## Limitations

Long-term unreduced posterior hip dislocation is a rare disease. We only examined 24 cases of long-term unreduced posterior hip dislocation; however, a total of 300 cases were examined. The sample size was small. When calculating experimental data, such as sensitivity and specificity, and other experimental data (especially specificity), an insufficient sample size might have resulted in misleading estimates of these indexes, especially overestimated specificity. Posterior hip dislocations cause shortening and internal rotation deformities of the affected limbs and cause postural scoliosis. Because the patients included in our experiment had varying degrees of dislocation and low total amounts, we did not record and analyze data such as the angle of scoliosis and the angle of internal rotation of the affected limb, although we recorded scoliosis and lower limb varus. A significant proportion of the patients with hip OA in the control group had bilateral OA, whereas the patients with long-term unreduced posterior hip dislocation were all unilateral, and the orthopedists who noted this inevitably led to biased experimental results.

## Conclusion

The results of the present study illustrate that the rotational center shift upside and the aureole sign on anteroposterior radiographs of the pelvis are important signs for differentiating long-term unreduced posterior hip dislocation from hip OA, and image of real acetabulum also has some diagnostic value. On CT examination, the rhombus sign and rotational center shift rearward on axial CT are very helpful in making the diagnosis of this disease, and the atrophic true acetabulum has not demonstrated a good diagnostic value because it is difficult to discern. All three CTs were of good value for the diagnosis of this disease, among which axial CT and coronal multiplanar reformation were of higher diagnostic value than sagittal multiplanar reformation.

## Data Availability

The original contributions presented in the study are included in the article/Supplementary Material; further inquiries can be directed to the corresponding authors.
